# Novel single‐operator through‐the‐scope traction device for endoscopic submucosal dissection: Outcomes of a multicenter randomized pilot ex‐vivo study in trainees with limited endoscopic submucosal dissection experience (with video)

**DOI:** 10.1002/deo2.174

**Published:** 2022-10-10

**Authors:** Dennis Yang, Hiroyuki Aihara, Muhammad K. Hasan, Cem Simsek, Hafiz Khan, Tony S. Brar, Venkata S. Gorrepati, Justin J. Forde, Kambiz Kadkhodayan, Mustafa A. Arain, Peter V. Draganov

**Affiliations:** ^1^ Center for Interventional Endoscopy AdventHealth Orlando USA; ^2^ Division of Gastroenterology Hepatology, and Endoscopy Brigham and Women's Hospital Harvard Medical School Boston USA; ^3^ Division of Gastroenterology and Hepatology University of Florida Gainesville USA

**Keywords:** endoscopic resection, endoscopic submucosal dissection, traction, trainees

## Abstract

**Objectives:**

Endoscopic submucosal dissection is a technically demanding procedure. The pilot study aimed to prospectively evaluate the efficacy and safety of a novel single‐operator through‐the‐scope dynamic traction device among trainees with limited endoscopic submucosal dissection (ESD) experience.

**Methods:**

Randomized, controlled, pilot study comparing traction‐assisted ESD (T‐ESD) versus conventional ESD (C‐ESD) in an ex‐vivo porcine stomach model. Trainees were randomized to group 1 (T‐ESD followed by C‐ESD) and group 2 (C‐ESD followed by T‐ESD). Lesions were created on the gravity‐dependent area of the stomachs. The primary outcome was submucosal dissection speed. Secondary outcomes included differences in en‐bloc resection, adverse events, and workload, assessed by the National Aeronautical and Space Administration Task Load Index (NASA‐TLX).

**Results:**

Five trainees performed two T‐ESD and two C‐ESD each, for a total of 20 procedures. Submucosal dissection speed was significantly faster in the T‐ESD group compared to the C‐ESD group (43.32 ± 22.61 vs. 24.19 ± 15.86 mm^2^/min; *p* = 0.042). En‐bloc resection was achieved in 60% with T‐ESD and 70% with C‐ESD (*p* = 1.00). The muscle injury rate was higher in the C‐ESD group (50% vs. 10%; *p* = 0.21) with 1 perforation reported with C‐ESD and none with T‐ESD. NASA‐TLX physical demand was lower with T‐ESD compared to C‐ESD (4.5 ± 2.17 vs. 6.9 ± 2.50; *p* = 0.03).

**Conclusion:**

T‐ESD resulted in faster submucosal dissection and less physical demand when compared to C‐ESD, as performed by trainees in an ex‐vivo gravity‐dependent model. Future studies are needed to assess its role in human ESD cases.

## INTRODUCTION

Endoscopic submucosal dissection (ESD) in the West has been restricted to specialized centers,^1^, ^2^, ^3^ primarily given the high technical demand, longer procedure time, and potential for higher risk of adverse events.^4^, ^5^ Maintaining adequate visualization of the dissection plane during ESD is often the rate‐limiting step and is regarded as one of the most difficult aspects of the procedure.^6^ Providing appropriate traction to expose the dissection field is crucial during ESD; however, accomplishing this feat often requires a deep understanding of advanced endoscopic resection principles. Furthermore, current strategies are limited by the ability to efficiently provide and readjust traction in multiple directions during real‐time ESD and/or the complexity of the traction device.^6^, ^7^, ^8^, ^9^, ^10^

A novel traction device (Tracmotion; Fujifilm, Lexington, MA, USA) for ESD was recently introduced and cleared by the US Food and Drug Administration. This traction device is a single‐operator, through‐the‐scope retraction device with 360° rotatable grasping forceps that enable tissue manipulation during ESD. In this randomized pilot ex‐vivo animal study, we aimed to evaluate the efficacy and safety of traction‐assisted ESD (T‐ESD) using this device compared to the conventional ESD (C‐ESD) technique when performed by trainees with limited ESD experience.

## METHODS

### Study outcome measures

The primary outcome was submucosal dissection speed between T‐ESD and C‐ESD. Secondary outcomes included differences in procedure times, resection outcomes, adverse events (e.g., specimen injury, muscle injury, and perforation), and physical/mental workload on the participants.

### Study design

This was a multicenter randomized, controlled, pilot study comparing T‐ESD versus C‐ESD in an ex‐vivo porcine stomach model among five trainees with limited ESD experience. The five trainees were advanced endoscopy fellows from three different institutions. The five trainees were randomized to one of two groups. Group 1 performed T‐ESD followed by C‐ESD, and group 2 performed C‐ESD followed by T‐ESD. The study was approved by the Institutional Review Board at the participating centers, with AdventHealth Orlando serving as the coordinating center.

### Study participants

All five participants had limited experience with C‐ESD, defined as having performed fewer than 10 ex‐vivo C‐ESD procedures and less than two human cases. None of the trainees had prior exposure to the traction device. At the beginning of this study and prior to any procedures, participants were enrolled in two structured learning sessions. The first session consisted of a 45‐min lecture with videos on the steps in C‐ESD, including basic maneuvering of the endoscope, ESD knives, and electrocautery settings. Similarly, the participants underwent a 45‐min lecture with videos on methods to achieve traction in ESD, and a step‐by‐step guide on how to set up and use the dedicated ESD traction device. In addition, all the participants had a 45‐min hands‐on practice session performing T‐ESD on an inanimate model with supervision by an ESD expert (Dennis Yang, Hiroyuki Aihara, and Peter V. Draganov).

### Porcine stomach ex‐vivo model

Resected pig esophagus stomachs were used for this ex‐vivo study. Standardized lesion location and marking were performed as follows: the 3 cm distance from the retracted tip of the ESD knife to the shaft of the catheter was measured with a ruler and marked with adhesive tape. The endoscope was then inserted into the explanted esophagus and advanced into the stomach. Using the ESD knife, a thermal cautery mark was placed at 12 o'clock. The ESD knife was then advanced out of the endoscope until the 3 cm mark on the shaft of the catheter could be visualized endoscopically. By placing the tip of the ESD knife at the site of the first cautery mark, we estimated where to place the second cautery mark in the 6 o'clock position, 3 cm proximal from the first thermal marking. Cautery marks were then made at the 9 and 3 o'clock positions in a similar fashion, followed by completion of the circular‐shaped lesion by placing cautery marks spaced approximately 3 mm from each other. The first lesion was created in the proximal stomach approximately 10 cm distal to the gastroesophageal junction and the second one more distally in the antrum. Both lesions were created on the gravity‐dependent side of the stomach. The decision was to create the lesion on the gravity‐dependent side of the stomach to eliminate the assistance of gravity when evaluating the effect of the traction device during ESD. A separate pig esophagus‐stomach explant was used by the participant for each arm in the study.

### Procedure

All procedures were supervised by an ESD expert (Dennis Yang, Hiroyuki Aihara, Muhammad K. Hasan, and Peter V. Draganov; Supplemental Video). Verbal guidance was provided, but no hands‐on direct technical assistance was given to the participants. All procedures were performed using a standard needle‐type knife (Flush Knife DK2620JI ‐B25; Fujifilm). The following setting was used for mucosal incision and submucosal dissection for all cases: EndoCut I 2‐3‐1, PreciseSect 5.6 (VIO 3; ERBE, Marietta, GA, USA).

#### C‐ESD

**FIGURE 1:**
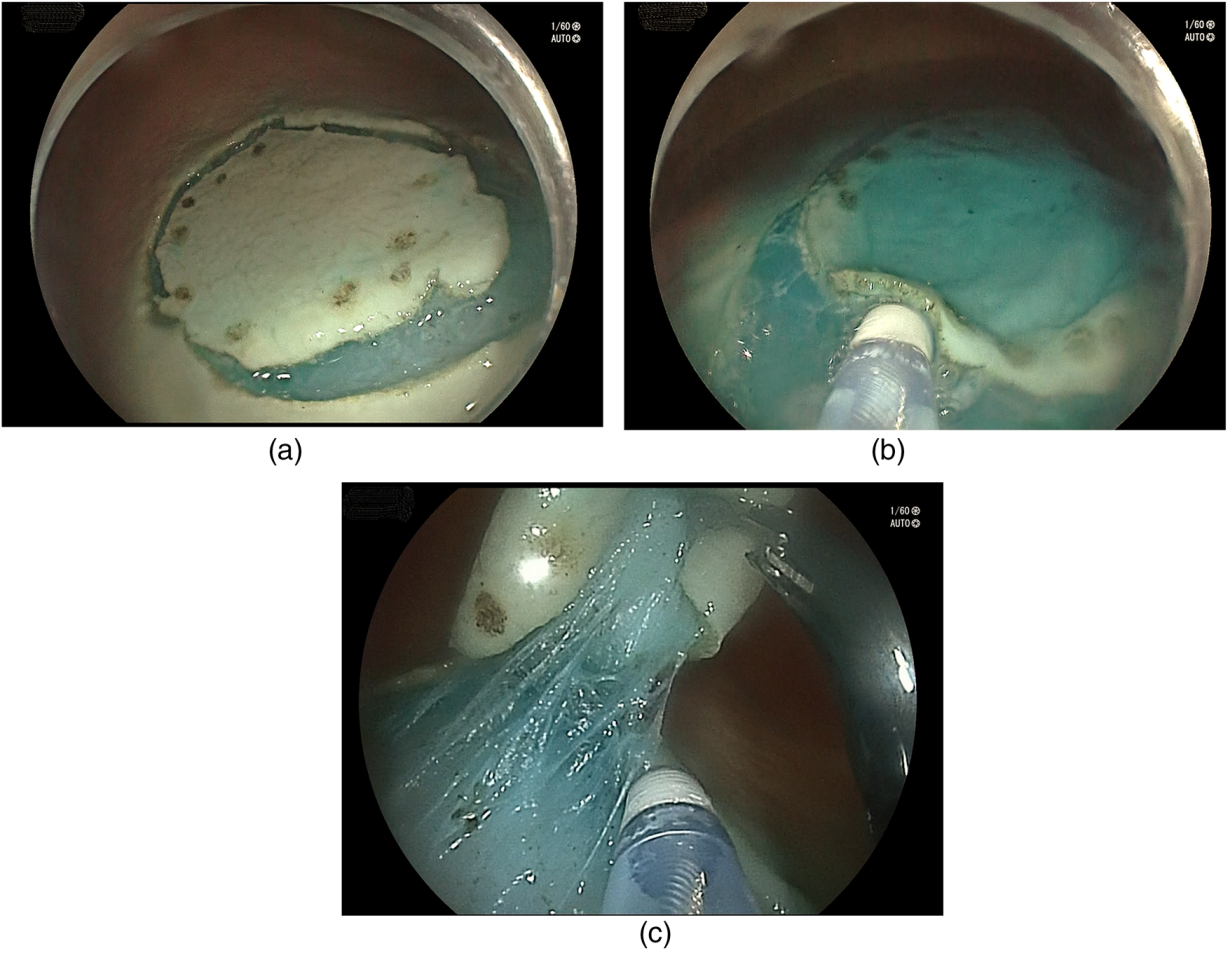
Circumferential mucosal incision was completed prior to submucosal dissection in both arms of the study (a). Conventional endoscopic submucosal dissection (C‐ESD) (b) and traction‐assisted ESD (c).

The procedure was performed using a single‐channel diagnostic upper endoscope (EG‐ 760R; Fujifilm) with a distally fitted transparent cap (Olympus America, Center Valley, PA). C‐ESD was performed as previously described.^11^ First, the submucosal injection was performed with a solution of saline and methylene blue using a standard injection needle. Subsequent submucosal injections were performed with either the injection needle or the needle‐type knife when necessary. Following submucosal lifting, a full circumferential mucosal incision was completed prior to submucosal dissection for lesion removal (Figure 1).

#### ESD traction device

**FIGURE 2:**
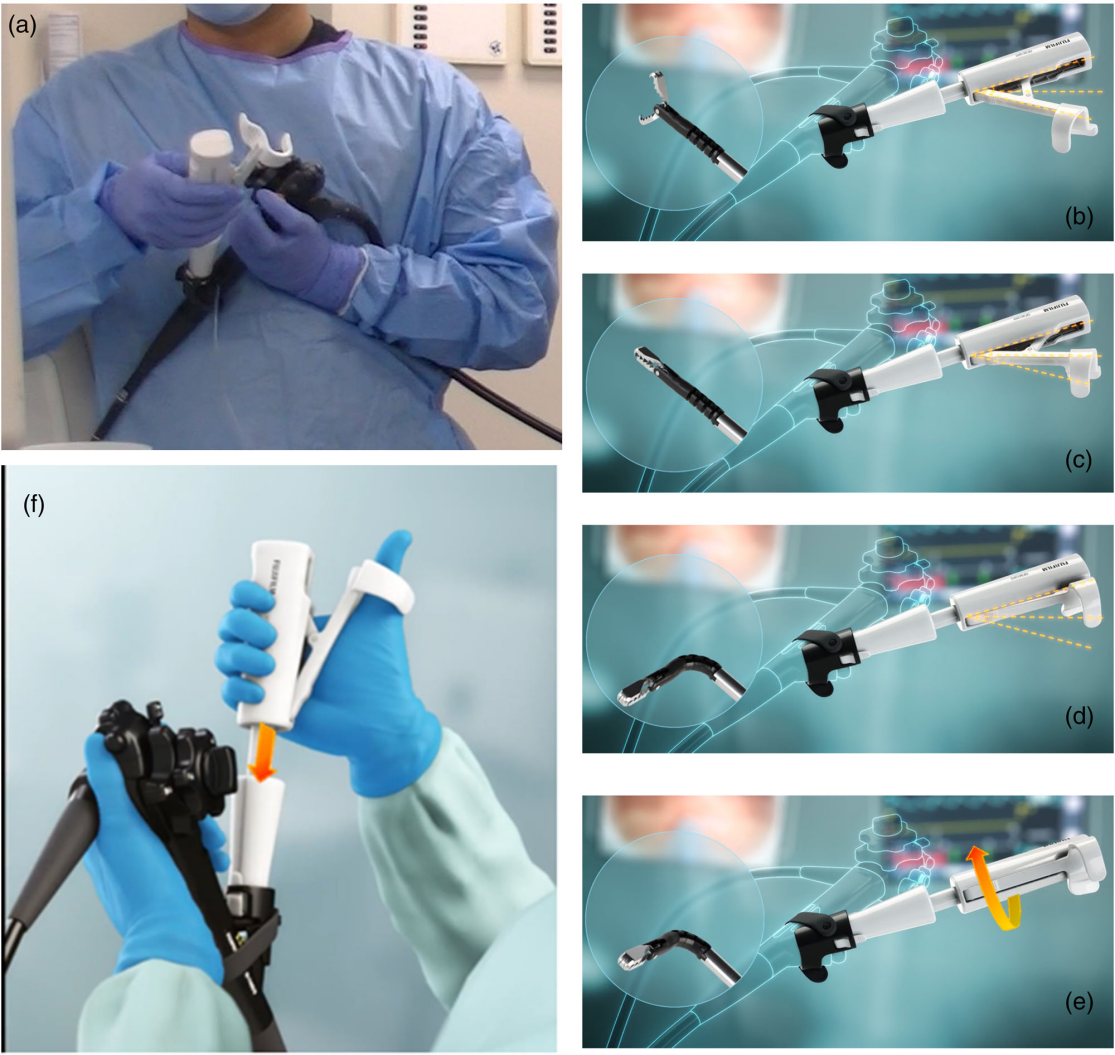
The traction device used in this study (Tracmotion; Fujifilm, Lexington, MA, USA) is a single operator, a through‐the‐scope device with 360° rotatable grasping forceps that can be opened and closed repeatedly to allow tissue manipulation and traction during submucosal dissection (a). The opened handle position opens the jaws of the grasping forceps (b). Closing the handle halfway results in the closure of the jaws of the grasping forceps (c). A fully closed handle bends the grasping forceps towards a 90‐degree angle (d). Rotation of the handle translates into rotation of the grasping forceps (e). Pushing down on the handle (arrow) further extends out the grasping forceps (f).

The Tracmotion device is a single‐operator, through‐the‐scope traction device designed for ESD (Figure 2). It consists of 360° rotatable grasping forceps that can be opened and closed repeatedly to allow tissue manipulation and traction during submucosal dissection. The device requires a double channel endoscope with a 3.7 mm or larger instrument inner channel diameter and a working length of 1030 mm.

#### Traction‐assisted ESD

For T‐ESD, initial submucosal lifting and circumferential mucosal incision were performed in a similar manner as in the C‐ESD group. Upon completion of the mucosal incision, the single‐channel upper endoscope (EG‐760R; Fujifilm) was exchanged for the double‐channel endoscope (EI‐740D; Fujifilm) with the mounted traction device. T‐ESD was then performed with the needle knife with the traction device grasping forceps providing traction during the procedure.

### Data collection and definitions

#### Procedural characteristics

The total injection volume (ml) was recorded. Upon completion of the procedure, the specimen size was calculated by measuring the long and short axis of the resected specimen. In cases where complete resection was not achieved, the submucosal dissection area was calculated. To calculate the submucosal dissection area, the pig stomach was excised to access the ESD resection bed. Using a ruler, we measured the long and short axis of the resection bed.

#### Procedure times

For all procedures, time was recorded separately. Total procedure time was defined as the time from scope insertion to final scope withdrawal from the explant. If the total procedure time reached 1 h, the procedure was considered as failed (incomplete resection) and aborted. Mucosal incision time was defined as the time from the initial submucosal injection to the time the circumferential incision was completed. Submucosal dissection time was defined as the time from which submucosal dissection started to the time the simulated lesion was completed and resected or to the time when the 1 h total procedure time had been reached. For the T‐ESD group, we also measured (1) scope exchange time, defined as the time from withdrawal of the single channel endoscope to insertion of the double‐channel endoscope; and (2) traction device set‐up time, defined as the time from insertion of the traction device into the working channel of the endoscope to the time when the grasping forceps were visualized endoscopically.

#### Resection outcomes

Complete resection was defined as the removal of the simulated lesion within 1 h of total procedure time. En‐bloc resection was defined as the successful removal of the specimen in one piece. Specimen injury was defined as any visible injury to the simulated lesion within the circumferential cautery marks (e.g., cutting into the lesion within the cautery marks, puncturing through the lesion). Muscle injury was defined as any visible defect to the muscle layer but without full‐thickness injury, whereas perforation was defined as any full‐thickness defect at the resection bed.

#### Mental and physical workload

After completing each procedure, participants graded procedural difficulty using the National Aeronautical and Space Administration (NASA) Task Load Index (TLX), a quantitative scoring system developed and validated by NASA to evaluate the workload in a procedure.^12^ There are six factors on the NASA‐TLX: mental demand, physical demand, temporal demand, performance, effort, and frustration. Participants rated each factor based on a 10‐point visual analog scale, specifying their level of agreement with each statement by indicating a position on the scale, from 0 (very low) and 10 (very high).

### Statistical analysis

Descriptive statistics for each continuous variable were obtained and expressed as mean ± standard deviation. Categorical variables were reported using proportion (%). To calculate specimen size and submucosal dissection area, the equation area = π × (long axis × short axis)/2 was used. In cases where complete resection was achieved, then specimen size and submucosal dissection area were considered equivalent. Submucosal dissection speed was calculated as follows: (submucosal dissection area in mm^2^/submucosal dissection time in min). An average of all NASA‐TLX factor scores was calculated to derive the mean total NASA‐TLX score. Chi‐square or Fisher's exact test for categorical variables and the *t‐*test for continuous variables were performed to compare outcomes between the two groups. Nominal *p*‐values are reported; *p*‐values < 0.05 were considered significant. Statistical analyses were performed using SAS (SAS Institute, Cary, NC). The Research Randomizer (available at www.randomizer.org) was used for randomization in this study.

## RESULTS

### T‐ESD versus C‐ESD techniques

#### Procedural characteristics

After randomization, three participants performed two T‐ESDs followed by two C‐ESDs, and two participants performed two C‐ESDs followed by two T‐ESDs. All five participants performed a total of four ESDs, for a total of 20 procedures.

Procedural characteristics are summarized in Table 1. Similar total volumes of injectate for submucosal lifting were used in both groups, even though the submucosal dissection area was larger in the T‐ESD group compared to the C‐ESD group (110.02 ± 54.48 vs. 66.89 ± 34.53 mm^2^; *p* = 0.048). The mean resected specimen size was 134.35 ± 54.86 and 78.19 ± 27.58 mm^2^ in the T‐ESD and C‐ESD groups, respectively (*p* = 0.009).

**TABLE 1:** Comparison of procedural characteristics between traction‐assisted endoscopic submucosal dissection (T‐ESD) versus conventional ESD (C‐ESD)

Variable	T‐ESD	C‐ESD	*p*‐value
Injection volume (ml)	65.3 ± 50.31	72.5 ± 53.39	0.76
Specimen size (mm^2^)	134.35 ± 54.86	78.19 ± 27.58	0.009
Submucosal dissection area (mm^2^)	110.02 ± 54.48	66.89 ± 34.53	0.048
Mucosal incision time (min)	16.42 ± 9.15	14.23 ± 13.25	0.67
Scope exchange time (min)	1.74 ± 0.99	N/A	–
Submucosal dissection time (min)	26.56 ± 10.68	31.26 ± 11.88	0.36
Traction device set‐up time (min)	1.06 ± 0.83	N/A	–
Total procedure time	55.43 ± 14.09	48.49 ± 15.40	0.31
Submucosal dissection speed (mm^2^/min)	43.32 ± 22.61	24.19 ± 15.86	0.042

Abbreviations: C‐ESD, conventional endoscopic submucosal dissection; T‐ESD, traction‐assisted ESD.

#### Procedure times

There was no difference in mean total procedure time between the T‐ESD and C‐ESD groups (55.43 ± 14.09 vs. 48.49 ± 15.40; *p* = 0.31). Similarly, mean mucosal incision and submucosal dissection times were similar between the two groups (Table 1).

#### Submucosal dissection speed

The mean submucosal dissection speed was significantly faster in the T‐ESD group (43.32 ± 22.61 mm^2^/min) than in the C‐ESD group (24.19 ± 15.86 mm^2^/min; *p* = 0.042).

#### Resection outcomes

Complete resection was achieved in 60% (6/10) of the T‐ESD group and 70% (7/10) of the C‐ESD group (*p* = 1.00; Table 2). All cases of incomplete resection were due to the inability to completely remove the simulated lesion within 1 h. En‐bloc resection was achieved in all cases of T‐ESD (6/6; 100%) and C‐ESD (7/7; 100%). When compared to the T‐ESD group, there was a trend toward a higher muscle injury (50% vs. 10%; *p* = 0.14) and specimen injury (30% vs. 0; *p* = 0.21) rate in the C‐ESD group; but these were not statistically different. There were no perforations in the T‐ESD group versus one identified in the C‐ESD group.

**TABLE 2:** Comparison of resection outcomes between traction‐assisted endoscopic submucosal dissection versus conventional ESD

Variable	T‐ESD	C‐ESD	*p*‐value
Complete resection ≤ 60 min	6/10 (60)	7/10 (70)	1.00
En‐bloc resection	6/6 (100)	7/7 (100)	1.00
Muscle injury	1/10 (10)	5/10 (50)	0.14
Specimen injury	0	3 (30)	0.21
Perforation	0	1 (10)	1.00

Abbreviations: C‐ESD, conventional endoscopic submucosal dissection; T‐ESD, traction‐assisted ESD.

#### Mental/physical workload

Each participant evaluated their workload during each ESD procedure using the NASA‐TLX to assess mental and physical workload (Table 3). The total NASA‐TLX score was similar in both groups (36.45 ± 11.13 with T‐ESD vs. 36.2 ± 16.11 with C‐ESD; *p* = 0.92). When the score was subdivided into individual components, the score for physical demand was significantly lower in the T‐ESD group compared with the C‐ESD group (4.5 ± 2.17 vs. 6.9 ± 2.50; *p* = 0.03).

**TABLE 3:** Comparison of National Aeronautical and Space Administration Task Load Index scores between traction‐assisted endoscopic submucosal dissection versus conventional ESD

NASA‐TLX	T‐ESD	C‐ESD	*p*‐value
Cumulative score	36.45 ± 11.13	36.2 ± 16.11	0.92
Mental demand	6.65 ± 2.52	6.4 ± 2.95	0.83
Physical demand	4.5 ± 2.17	6.9 ± 2.50	0.03
Temporal demand	6.6 ± 2.40	5.45 ± 3.01	0.36
Performance	5.25 ± 3.10	5.15 ± 3.79	0.95
Effort	7.4 ± 1.41	6.9 ± 2.44	0.58
Frustration	6.05 ± 2.75	5.45 ± 3.33	0.67

Abbreviations: C‐ESD, conventional endoscopic submucosal dissection; NASA‐TLX, National Aeronautical and Space Administration Task Load Index; T‐ESD, traction‐assisted ESD.

## DISCUSSION

This multicenter randomized pilot study demonstrated that T‐ESD with a novel single‐operator through‐the‐scope traction device significantly increased submucosal dissection speed and reduced physical demand among endoscopists with limited experience with ESD. T‐ESD trended towards improved safety when compared to C‐ESD.

ESD is fundamentally challenging because of the level of technical proficiency required to maintain visualization of the submucosal dissection plane during the procedure.^6^ Loss of visualization of the dissection plane increases the risk of adverse events and leads to unfavorable resection outcomes. Intuitively, effective countertraction methods to improve visualization during ESD should improve procedural safety and reduce technical demand. The ideal traction device should be easy to operate and permit dynamic adjustment of traction during ESD, independently of endoscope movement. The novel traction device used in this study consists of rotatable grasping forceps that can be opened and closed repeatedly to allow real‐time tissue manipulation and adjustment of traction during the procedure. Our study demonstrated that T‐ESD resulted in a significantly faster submucosal dissection time when compared to C‐ESD (43.32 ± 22.61 mm^2^/min vs. 24.19 ± 15.86; *p* = 0.042). Hence, one might assume that larger areas resected with the T‐ESD would lead to even better results given that submucosal dissection involves the main component of the procedure. Additionally, by facilitating exposure to the submucosal dissection plane, T‐ESD potentially increases the safety profile of ESD among learners, as evidenced by the trend toward a lower rate of muscle injury in the T‐ESD group as compared with the C‐ESD group (10% vs. 50%; *p* = 0.14). Furthermore, there were no cases of specimen injury or perforation in the T‐ESD group, whereas three (30%) and one (10%) were reported with C‐ESD, respectively.

Our study included endoscopists with limited ESD experience. Based on the NASA‐TLX scores, trainees in our study indicated significantly lower physical demand with T‐ESD when compared to C‐ESD, presumably because of more efficient submucosal dissection and visualization when utilizing the traction device. Notably, the trainees in this study had no prior experience with this novel traction device, and thereby the learning phase associated with performing T‐ESD may have accounted for the lack of improvement in other NASA‐TLX parameters when compared to C‐ESD. Nonetheless, our findings are consistent with a recent study from Japan demonstrating improved resection rates and dissection speed of colorectal ESD among trainees when a traction device was introduced into their training program.^13^ In aggregate, our preliminary findings add to the mounting body of evidence that devices aiding with traction during ESD reduce the technical complexity of the procedure and are associated with improved outcomes. Specific to our study, this novel traction device may also have the added benefit of providing easy‐to‐operate dynamic adjustments during the procedure, which can be particularly helpful among trainees who are not yet familiar with the concepts of traction–countertraction.

There are several strengths to this pilot study. First, the study was randomized, and trainees were blinded to the exact outcome measures. Second, the simulated lesions were created on the dependent area in the ex‐vivo model to exclude the aid from gravity, allowing us to analyze the pure efficacy of the device in visualizing the dissection plane. However, the more difficult location did lead to higher incomplete resection rates by trainees with limited experience with traction and ESD.

This study is not without limitations. First, there were some baseline differences in terms of simulated lesion size between the T‐ESD and C‐ESD groups. Although the marking of the lesion was standardized for all the procedures, the overall resected specimen and submucosal dissection areas were significantly larger in the T‐ESD group when compared to the C‐ESD group. Potential explanations for this discrepancy include variability in lesion marking among the endoscopists (Dennis Yang, Hiroyuki Aihara, and Peter V. Draganov) despite the standard approach and trainees dissecting significantly more lateral than the thermal markings around the simulated lesion. Nonetheless, we accounted for this difference by calculating the dissection speed (mm^2^/min) in addition to procedural times. An alternate explanation is that the submucosal dissection and resection area could have increased from lesion manipulation with the traction device. However, we believe this to be unlikely, given that circumferential mucosal incision had already been performed prior to T‐ESD. Irrespectively, we should acknowledge that an inadvertent larger resection area could potentially prolong total procedure time and theoretically increase the risk of adverse events, including delayed bleeding. Importantly, we recognize that the difference in simulated lesion size between the two groups limits the interpretability of our preliminary results and additional studies will be needed to better establish the impact of T‐ESD. Second, we acknowledge that intraprocedural bleeding during ESD cannot be evaluated in an ex‐vivo model. Nonetheless, improved visualization of the submucosal layer via traction would intuitively facilitate the identification and treatment of bleeding vessels. Third, the study was designed to compare T‐ESD to C‐ESD. Hence, our results do not provide insight into how this traction device compares with other currently available traction techniques for ESD. Furthermore, while isolating the simulated lesions to the gravity‐dependent portion of the stomach allowed us to specifically evaluate the added benefit of a traction device during ESD, this strategy may have artificially augmented the outcomes of T‐ESD as compared to C‐ESD. In a true clinical scenario, additional interventions, including repositioning of the lesion on the anti‐gravity side and/or using additional techniques/devices (i.e., traction methods, a longer distal attachment hood) would have most likely been implemented for C‐ESD to potentially improve efficiency and safety. Yet, these were not employed in this study to minimize potential variables on performance. Lastly, because this was a pilot study, the overall number of participants and cases was relatively low. Hence, the lack of a statistically significant difference may not necessarily exclude the possibility of clinically relevant differences. Larger in vivo and human feasibility studies are needed to corroborate these initial results.

In conclusion, T‐ESD resulted in faster submucosal dissection and less physical demand when compared to C‐ESD, as performed by trainees in an ex‐vivo gravity‐dependent model. Future studies are warranted to validate these preliminary findings and to evaluate the role of this ESD traction device in human cases.

## CONFLICT OF INTEREST

Dennis Yang is a consultant for Olympus, Fujifilm, Apollo Endosurgery, Medtronic, and Microtech. Hiroyuki Aihara is a consultant for Olympus, Fujifilm, Boston Scientific, Medtronic, and 3D Matrix. Kambiz Kadkhodayan has no disclosures. Mustafa A. Arain is a consultant for Boston Scientific, Olympus, Cook Medical, and Medtronic. Muhammad K. Hasan is a consultant for Boston Scientific and Olympus. Peter V. Draganov is a consultant for Olympus, Boston Scientific, Fujifilm, Cook Medical, and Medtronic. All other authors have nothing to disclose. The authors received no financial incentive, including no consulting fees, relating to this study or the devices used in this study.

## FUNDING INFORMATION

There was no funding for this study. Fujifilm loaned the endoscopic equipment and provided the devices and ex‐vivo models free of charge used in this study. ERBE loaned the use of five electrocautery generators for this study.

## Supporting information

Video 1
